# DNA Methylation Modification Map to Predict Tumor Molecular Subtypes and Efficacy of Immunotherapy in Bladder Cancer

**DOI:** 10.3389/fcell.2021.760369

**Published:** 2021-12-03

**Authors:** Fangdie Ye, Yingchun Liang, Jimeng Hu, Yun Hu, Yufei Liu, Zhang Cheng, Yuxi Ou, Chenyang Xu, Haowen Jiang

**Affiliations:** ^1^ Department of Urology, Huashan Hospital, Fudan University, Shanghai, China; ^2^ Fudan Institute of Urology, Huashan Hospital, Fudan University, Shanghai, China; ^3^ National Clinical Research Center for Aging and Medicine, Huashan Hospital, Fudan University, Fudan, China

**Keywords:** bladder cancer, DNA methylation regulators, immunotherapy, prognostic model, tumor microenvironment

## Abstract

**Background:** Considering the heterogeneity and complexity of epigenetic regulation in bladder cancer, the underlying mechanisms of global DNA methylation modification in the immune microenvironment must be investigated to predict the prognosis outcomes and clinical response to immunotherapy.

**Methods:** We systematically assessed the DNA methylation modes of 985 integrated bladder cancer samples with the unsupervised clustering algorithm. Subsequently, these DNA methylation modes were analyzed for their correlations with features of the immune microenvironment. The principal analysis algorithm was performed to calculate the DMRscores of each samples for qualification analysis.

**Findings:** Three DNA methylation modes were revealed among 985 bladder cancer samples, and these modes are related to diverse clinical outcomes and several immune microenvironment phenotypes, e.g., immune-desert, immune-inflamed, and immune-excluded ones. Then patients were classified into high- and low-DMRscore subgroups according to the DMRscore, which was calculated based on the expression of DNA methylation related genes (DMRGs). Patients with the low-DMRscore subgroup presented a prominent survival advantage that was significantly correlated to the immune-inflamed phenotype. Further analysis revealed that patients with low DMRscores exhibited less TP53 wild mutation, lower cancer stage and molecular subtypes were mainly papillary subtypes. In addition, an independent immunotherapy cohort confirmed that DMRscore could serve as a signature to predict prognosis outcomes and immune responses.

**Conclusion:** Global DNA methylation modes can be used to predict the immunophenotypes, aggressiveness, and immune responses of bladder cancer. DNA methylation status assessments will strengthen our insights into the features of the immune microenvironment and promote the development of more effective treatment strategies.

## Introduction

DNA methylation modification is one of the most representative epigenetic modifications, which is indispensable in vertebrate development and illnesses (Ortega-Recalde and Hore, 2019; da Rocha and Gendrel, 2019). Besides, it has been demonstrated to associate with multiple biological functions in cancer, e.g., the formation and evolution of tumor microenvironment, as well as impairment restoration in the immune cycle (Chen et al., 2020; Zhang et al., 2020a). On the other hand, abnormal DNA methylation is also significantly related to the occurrence of multiple cancer types, such as sarcoma (Koelsche et al., 2021), bladder cancer (Liu et al., 2021a), and vulvar intraepithelial neoplasia (Thuijs et al., 2021).

Bladder cancer is an extremely malignant urogenital neoplasm (Siegel et al., 2021). Heterogeneous distributions of genome clusters lead to molecular and cellular heterogeneity in tumors, which affect clinical outcomes and treatment responses (Qiu et al., 2019; Craig et al., 2020). Despite pronounced progress in the treatment of bladder cancer, more effective therapeutic strategies are still in demand. Studies have demonstrated that several genes involved in the occurrence and progression of bladder cancer are regulated by promoter methylation. For example, Chen X et al. built a diagnostic model based on 2 DNA methylation markers for early detection and recurrent monitoring of bladder cancer. Wilhelm CS et al. discovered that LINE1 hypomethylation may contribute to bladder cancer tumorigenesis, especially in women (Wilhelm et al., 2010). Kandimalla R et al. summarized the biomarkers of DNA methylation and identified that methylated genes, including SFRP1, SOX9, FHIT, CDH1, PMF1, RUNX3, LAMC2, and RASSF1A, are related to the poor clinical outcomes in bladder cancer patients (Kandimalla et al., 2013). In short, DNA methylation is involved in carcinogenesis or tumor inhibition across varying scenarios.

In recent years, immune checkpoint blockade therapy has emerged as a promising therapeutic strategy. It aims to enhance the immune activity of T lymphocytes to kill tumor cells by inhibiting immune checkpoints, such as PD-1 and its ligand PD-L1 (Yi et al., 2018). Studies have shown that immunotherapy could improve clinical outcomes of numerous tumors, such as ovarian cancer (Wan et al., 2021), bladder cancer (Han et al., 2021), and colorectal cancer (Liu et al., 2021b). However, patients respond to immunotherapy differently, and the effective rate of immune checkpoint blockade therapy has been less than 20%. The expression level of PD-L1, tumor microenvironment (TME), and tumor mutation burden (TMB) have been reported as signatures to evaluate the clinical responses to immunotherapy (Samstein et al., 2019). Previous studies demonstrated that DNA methylation may contribute to the alteration of TME. For instance, Sasidharan Nair V et al. discovered that DNA hypomethylation shall alter the expression of CTLA-4, TIGIT, and PD-1 genes (Sasidharan Nair et al., 2018). Elashi AA et al. also revealed that an abnormal promoter methylation profile is correlated to the peripheral upregulation of TIGIT and PD-1 in many cancers. They speculated that a combined administration of anti-PD-1 agents and demethylation inhibitors could be a more effective immunotherapeutic strategy than the current ones (Elashi et al., 2019). However, the regulatory mechanisms of global DNA methylation on tumor microenvironment and immune response in bladder cancer remain unclear.

In this study, genomic data and clinical information of 985 samples from six independent bladder cancer cohorts were included. DNA methylation modes were clarified by analyzing the expression of fifteen DNA methylation regulators in these samples We investigate the DNA methylation regulators rather than DNA methylation itself, because the biology function of DNA methylation would be altered according to the genomic environment. Specifically, three DNA methylation modes were identified to meet the criteria of immune-desert, immune-inflamed, and immune-excluded immunophenotypes, respectively. Moreover, an evaluation system was built to qualify the DNA methylation modes in individual patients, and the patients’ clinical responses to immunotherapy were assessed based on their DMRscore. Our study provides a new perspective to observe the global DNA methylation status and the immunophenotype of individual tumors in bladder cancer so that more specified precision medicine could be achieved.

## Results

### The Landscape of DNA Methylation Regulators in Bladder Cancer

**FIGURE 1:**
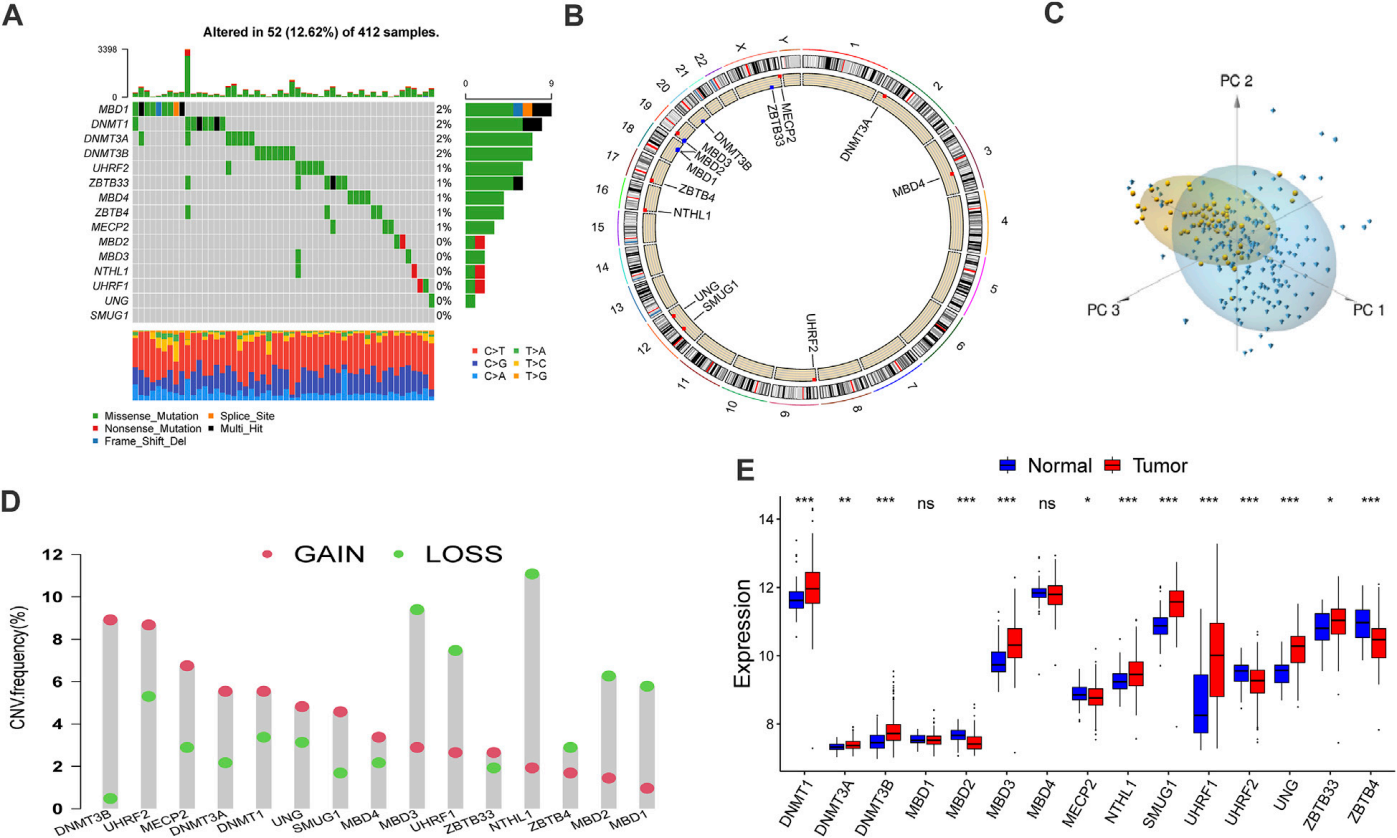
Landscape of genetic alteration and transcriptome variation of DNA methylation regulators in bladder cancer. **(A)** The alteration frequency of 15 DNA methylation regulators in 412 bladder cancer samples (TCGA-BLCA). the annotation of each variant types was displayed by the bagplots right barplots. Each cohort represented an individual sample. The stacked barplot below displayed conversion ratio for each sample. **(B)** The location of CNV alteration of DNA methylation regulators on 23 chromosomes was displayed by circular plot. **(C)** Principal component analysis (PCA) for the transcriptome characteristics of 15 DNA methylation regulators to distinguish tumors from normal samples in GSE13507 cohort. Tumor samples were labeled with blue color and normal samples were labeled with yellow. **(D)** The frequency of copy number variation in TCGA-BLCA cohort. Deletion frequency: the green dot and amplification frequency: red dot. The number represented the variation frequency. **(E)** The transcriptome characteristics of 15 DNA methylation regulators between normal and bladder cancer tissues. Tumor: red box; Normal: blue box. The median value: black lines in boxes, the outliers: black dots out boxes. The asterisks represented the statistical *p* value (*p < 0.05; **p < 0.01; ***p < 0.001).

We executed systematic research that included 15 DNA methylation regulators and summarizes the mutation rates of all these regulators in bladder cancer. Among 412 samples, 52 samples experienced alteration of DNA methylation regulators, with frequency 12.62%. According to the waterfall diagram, alterations of the MBD1 gene were the most frequent, and these alterations have been reported to participate in tumorigenesis. Besides, DNMT1, DNMT3A, and DNMT3B genes also exhibited an alteration frequency of 2% (Figure 1A). Furthermore, co-occurrence mutation was observed in several DNA methylation regulators despite their functional differences, including NTHL1, MBD3, MECP2, UHRF2, and ZBTB33 (Supplementary Figure S1C).

In addition, a prevalent CNV alteration was observed in the fifteen regulators (Figure 1D). Specifically, DNMT3B, UHRF2, and MECP2 demonstrated a widespread frequency of amplification in samples while MBD3, UHRF1, and NTHL1 were frequently detected. The locations and circle sequences of the DNA methylation regulators along the chromosomes are depicted in Figure 1B. Moreover, the principal component analysis revealed that the bladder cancer can be distinguished from normal samples by observing the expression levels of the 15 regulators (Figure 1C). In addition, the mRNA expressions of the 15 DNA methylation regulators are also significantly different between BLCA tumors and normal tissues (Figure 1E). In a word, the genomic imbalance of DNA methylation regulators is vital for bladder cancer tumorigenesis and development.

### DNA Methylation Regulator Clusters

**FIGURE 2:**
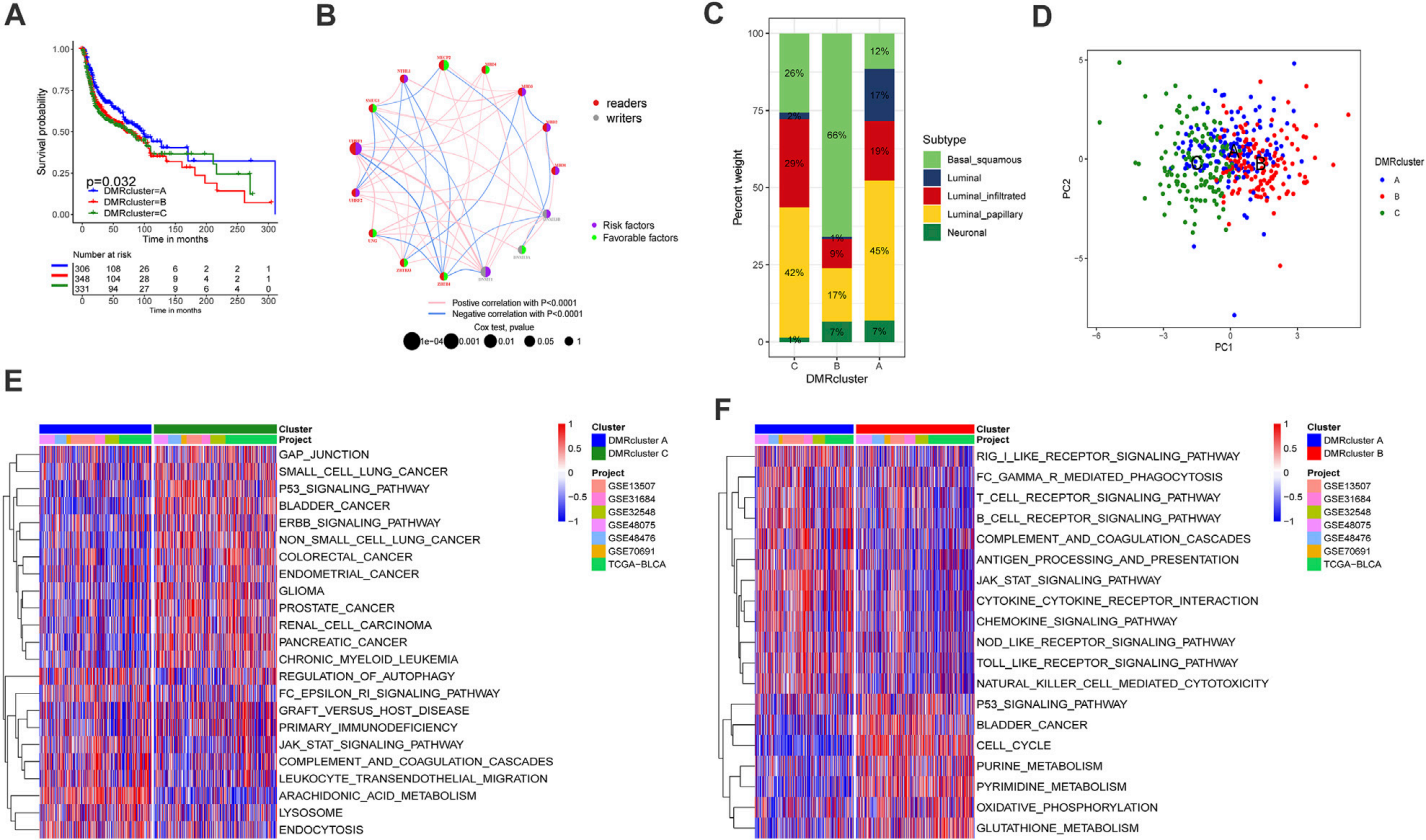
Clusters of DNA methylation modes and biological profiles of each cluster. A Kaplan-Meier curve with *p* value 0.032 displayed a remarkable difference among three DNA methylation modes, the DMRcluster B presented a remarkable poor clinical outcome. DMRcluster **(A)**: 306 samples, DMRcluster **(B)**: 348 samples and DMRcluster **(C)**: 331 samples. The meta cohort including 985 samples (GSE13507, GSE31684, GSE32548, GSE48075, GSE48476, GSE80691 and TCGA-BLCA). **(B)** The interplay among DNA methylation regulators in bladder cancer. Red and gary represented readers and writers respectively. The size of circles displayed the influence of each regulator on clinical outcomes. The lines connecting regulators represented their interactions, and thickness represented the correlation strength. Negative correlation was labeled with blue and positive correlation was labeled with red. Risk factor: purple, favorable factor: green. **(C)** The proportion of molecular subtypes in the three DNA methylation modes (TCGA-BLCA). Basal squamous subtype, green; Luminal subtype, blue; luminal infiltrated subtype, red; luminal papillary subtype, yellow; and Neuronal subtype, olivedrab. **(D)** Principal component analysis (PCA) for the transcriptome characteristics of three DMRclusters. DMRcluster A was labeled with blue color, DMRcluster **(B)** was labeled with red color and DMRcluster **(C)** was labeled with green. **(E,F)** Gene set variant analysis displayed the activation status of biological pathways in diverse DMRclusters. The heatmap help us to observe the difference of biology pathway activity among three DMRclusters. Blue represented inhibited pathways and red represented activated pathway. **(E)** DMRcluster **(A)** vs. DMRcluster **(B)**; F DMRcluster **(B)** vs. DMRcluster **(C)**.

The complete clinical information and transcriptome data of six GEO datasets (GSE13507, GSE31684, GSE32548, GSE48075, GSE48476, GSE80691) and TCGA-BLCA were enrolled into one cohort for further exploration. A univariate Cox regression analysis was performed to find the prognostic value of the 15 DNA methylation regulators in bladder cancer patients (Supplementary Figure S1B). Following that, the comprehensive landscape of the regulators’ intercorrelation and their prognostic attributes for bladder cancer were calculated by network planning (Figure 2B). From these results, we speculated that DNA methylation regulators may be related to the heterogeneity of bladder cancer. Therefore, unsupervised clustering was performed to explore ultramodern DNA methylation regulator clusters (DMRclusters) based on the expression levels of the regulators in the meta-cohort. Three DMRclusters were classified, including 306, 348, and 331 sample patients in DMRcluster A, B, and C, respectively, and these distinct DMRclusters could be distinguished via the principal component analysis (Figure 2D). Specifically, DMRcluster A presented a particularly prominent survival advantage, but DMRCluster B exhibited the worst clinical outcome in the integrated cohort (Figure 2A).

### The Immune Features of Distinct DMRclusters

**FIGURE 3:**
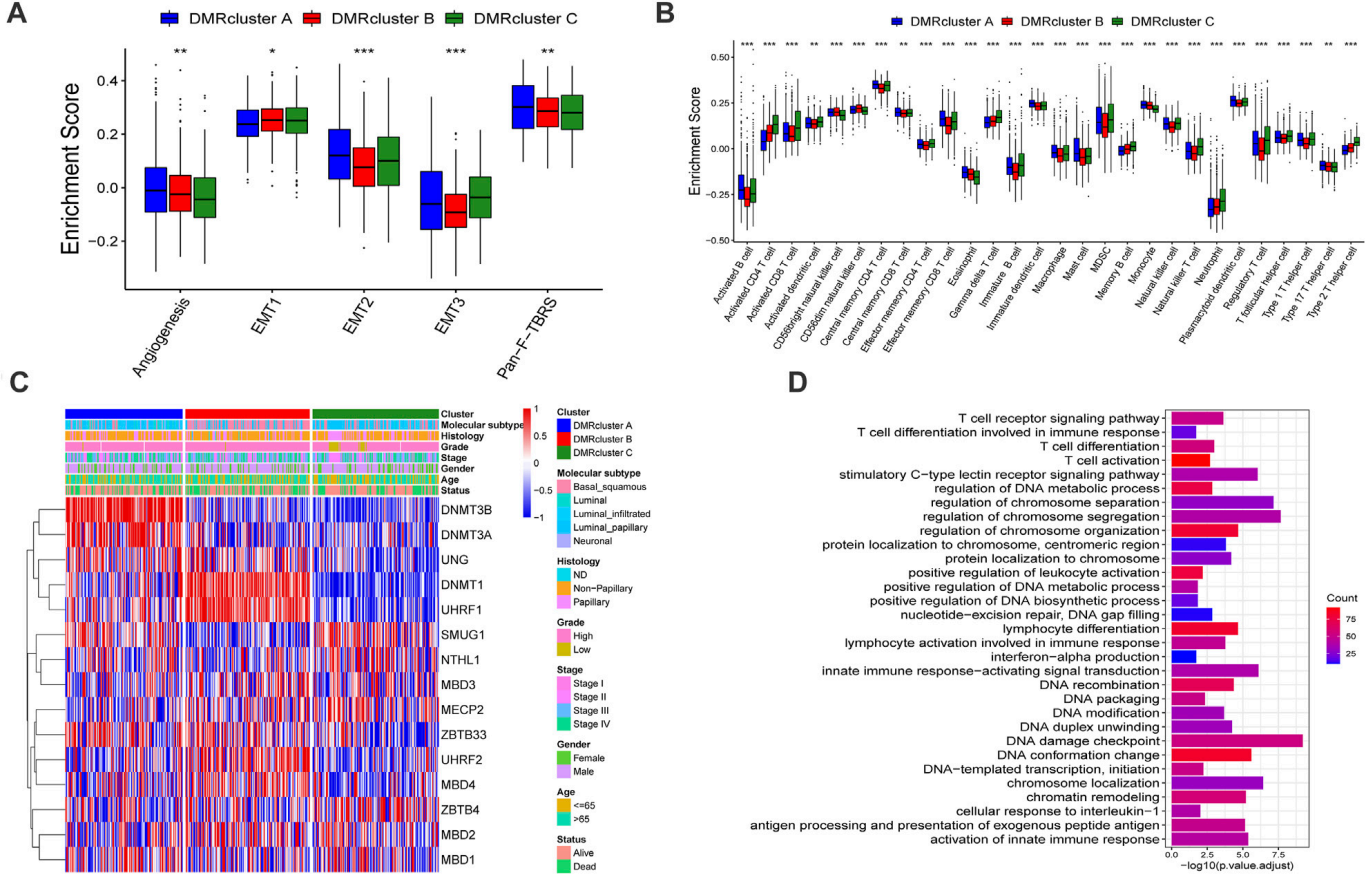
Tumor microenvironment characteristics and transcriptome profile in three DNA methylation modification modes. **(A)** stromal activation pathways among three different DNA methylation modification modes include EMT, angiogenesis and Pan-F-TBRS. **(B)** The content of each tumor microenvironment immune infiltrating cells in three DNA methylation modification modes. The median value: black lines in boxes, the outliers: black dots out boxes. **(C)** The heatmap help us to observe the expression level of DNA methylation regulators among different DMRclusters (TCGA-BLCA cohort). DMRcluster subtypes, Molecular subtypes, Histology, Grade, Stage, Gender, Age and Survival status were used as patient annotations. Red represented high expression level of DNA methylation regulators and blue represented low expression level. **(D)** Functional annotation for DNA methylation-related genes. Red represented more enriched genes and blue represented a small number of enriched genes.

The GSVA enrichment analysis was performed to identify the biological processes in the DMRclusters. Judging from the results, DMRcluster A was enriched in immune activation pathways, such as the T/B cell receptor signaling pathway, toll-like receptor signature, as well as complement and coagulation cascades. On the other hand, DMRcluster B was prominently associated with immune suppression, and DMRcluster C was even more prominent in carcinogenic pathways, including the P53 signature pathway and the ERBB signature pathway (Figures 2E,F). As expected, subsequent analyses revealed that DMRcluster A was significantly enriched in cells related to acquired immunity, including activated B cells, central memory CD4/CD8 T cells, and activated dendritic cells (Figure 3B). Such a finding could well explain the results of the survival analysis. Meanwhile, stromal activity (e.g., epithelial-mesenchymal transition &, EMT) was remarkably enriched in DMRcluster C (Figure 3A). Based on all the previous results, we speculated that these DMRclusters had remarkably diverse features in terms of immune cell infiltration into the tumor microenvironment. Specifically, DMRclusters A, B, C were featured by immune-inflamed, immune-desert, and immune-excluded phenotypes, respectively.

### The Transcriptome Data and Clinical Features of DMRclusters

**FIGURE 4:**
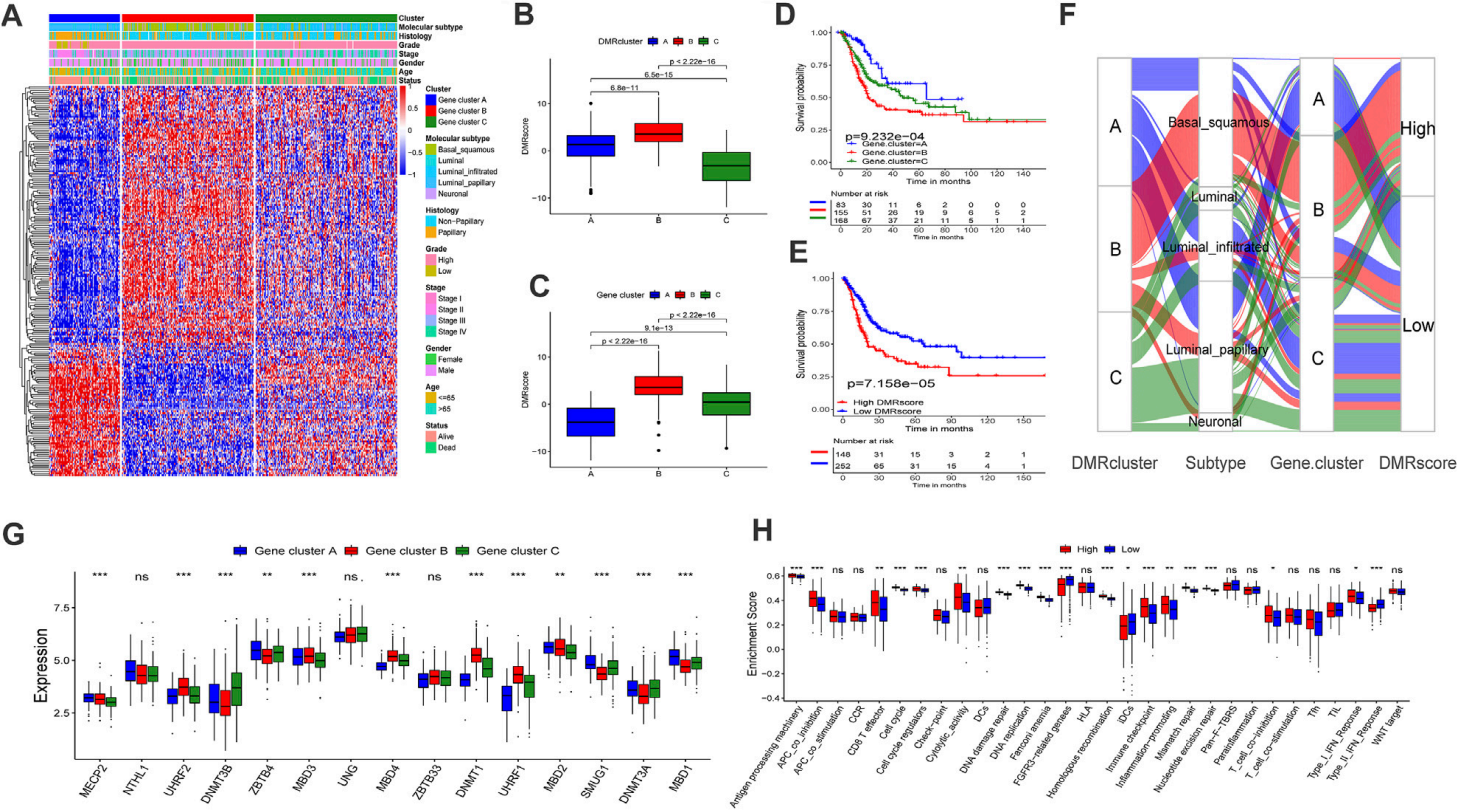
Construction of DNA methylation signatures for individual sample. **(A)** The heatmap help us to observe the transcriptome landscape among different Gene.clusters (TCGA-BLCA cohort). Gene.cluster subtypes, Molecular subtypes, Histology, Grade, Stage, Gender, Age and Survival status were used as patient annotations. Red represented high expression level and blue represented low expression level. B-C Differences in DMRscore among three DMRclusters **(B)** or Gene.clusters **(C)** in TCGA-BLCA cohort (Kruskal-Wallis test, *p* < 0.001). D-E Kaplan-Meier curve displayed a remarkable difference among three Gene. clusters (**(D)**, *p* < 0.001) or DMRscore subgroups (**(E)**, *p* < 0.001) in TCGA-BLCA cohort. **(F)** Sankey diagram displayed the alteration of DMRclusters, molecular subtypes, Gene.cluster and DMRscore. **(G)** The transcriptome characteristics of 15 DNA methylation regulators among Gene.clusters. Gene.cluster **(A)**: blue box; Gene.cluster **(B)**: red box. Gene.cluster C: green box. The median value: black lines in boxes, the outliers: black dots out boxes. **(H)** Differences in the known gene signatures between high DMRscore and low DMRscore subgroups. APC: antigen-presenting cells. The asterisks represented the statistical *p* value (*p < 0.05; **p < 0.01; ***p < 0.001).

To further investigate these DMRclusters in diverse biological processes and clinical features, we focused on the TCGA-BLCA cohort containing 407 bladder cancer patients and their exhaustive clinical information. Similarly, the patients were classified into three clusters with unsupervised clustering (Supplementary Figures S2A–D). Judging from the results DNA methylation regulators’ transcriptional profiles among the three DMRclusters demonstrated significant difference, which was validated by one-way ANOVA analysis (Supplementary Figure S2E). Specifically, DMRcluster A revealed high expression of DNMT3B and DNMT3A, DMRcluster B was characterized by higher DNMT1 and UHRF1 expressions, and DMRcluster C exhibited lower contents of DNMT1, DNMT3A, DNMT3B, and UHRF1 at various extents (Figure 3C). Patients with the luminal infiltrated subtype were characterized by DMRcluster A, while the basal squamous subtype was featured by DMRcluster B (Figure 2C); besides, both DMRclusters B and C were enriched in the neuronal subtype (Figure 4D). In bladder cancer treatments, the neuronal subtype is particularly difficult because of its poor clinical outcome, while the luminal papillary subtype is prone to better survival. Thus, we performed the K-M analysis, and the results also validated our conjecture that patients characterized by DMRclusters B and C exhibited significantly more rapid disease progression and poorer clinical outcomes, while DMRcluster A presented a remarkable survival advantage (Supplementary Figure S2F). In addition, the luminal infiltration subtype in bladder cancer is characterized by low tumor cell purity and high lymphocytic infiltration. Most patients with the luminal infiltration subtype were categorized into DMRcluster A, and only a small amount of luminal infiltration was observed in DMRcluster B (Figure 4D), suggesting that DMRcluster A is related to immune activation and DMRcluster B is associated with the immune-desert phenotype.

### Functional Annotations of DMRGs

To further explore the potential biological processes in each DMRcluster, the R package named “limma” was performed to find DMRGs, and a total of 832 genes were selected (Supplementary Figure S2G). GO analysis was executed on the DMRGs using the R package “clusterProfiler.” DMRGs were prominently enriched in immunity activation pathways, DNA methylation, and cell proliferation, which verified that DNA methylation is vital in the immune regulation of tumor progression (Figure 3D).

To further investigate the mechanisms of DNA regulation, the patients were classified into three genomic subtypes based on the expression of the 832 DMRGs. Similarly, the genomic subtypes were identified via the unsupervised clustering algorithm. They were termed Gene cluster A, B, and C, respectively (Supplementary Figures S3A–D), and they were all related to DNA methylation in bladder cancer. A heat map also demonstrated that the three Gene clusters can be distinguished by their signature transcriptomes (Figure 4A). According to the K-M survival method, Gene cluster A presented a remarkable survival advantage, while Gene cluster B was proved to be associated with a poorer prognosis (Figure 4D). Moreover, the three Gene clusters revealed significant differences in the expression of DNA methylation regulators (Figure 4G).

### TME Characteristics in the Three Gene.clusters

To identify the role of Gene clusters in the immune regulation of TME, we investigated the expression of cytokines and chemokines in Gene clusters. The targets of identification were chosen from the literature, among which ZEB1, TGFB2, PDGFRA, VIM, COL4A1, TGFBR2, TWIST1, ACTA2, and SMAD9 are related to transcripts of the transforming growth factor (TGF) b/EMT pathway. Besides, HAVCR2, CD80, LAG3, CD86, TIGIT, PDCD1, TNFRSF9, PD-L1, IDO1, CTLA4, and PD-L2 are associated with the transcripts of immune checkpoints, and CXCL10, PRF1, CD8A, CXCL9, GZMB, GZMA, TNF, IFNG, and TBX2 are associated with immune-activated transcripts (Sotiriou et al., 2006; Barbie et al., 2009; Ritchie et al., 2015; Zeng et al., 2019).

We found that the transcripts related to immune activation pathways were significantly up-regulated in Gene cluster B, but the patients in this cluster did not show an expected survival advantage. Previous studies revealed that high stromal activation was associated with limited immune activation (MacGregor et al., 2019). Therefore, we investigated the transcripts related to the (TGF)b/EMT pathway in this cluster and demonstrated stromal activation within. Based on these findings, we assumed that anti-tumor effects of immune cells in Gene cluster B are limited by stromal activation, indicating that Gene cluster B is the immune-excluded subtype. Besides, the transcripts of immune checkpoints were examined as highly expressed in Gene cluster B, suggesting that immunotherapy may bring unexpected outcomes (Supplementary Figures S3F–H).

To further investigate the functions of DMRGs, we examined the identified pathways in bladder cancer patients. Gene cluster A was found to enrich in CD8 T effector, DNA replication, mismatch repair, and antigen processing machinery pathways (Supplementary Figure S3E). Previous studies demonstrated that bladder cancer can be classified into five subtypes according to the molecular phenotype. Among them, the luminal-papillary subtype exhibits the best prognosis with a five-year survival rate of 60%. On the other hand, the five-year survival rate of neuronal bladder cancer is only 17% (Robertson et al., 2017). Our findings suggested that Gene cluster A was almost fully composed of the luminal-papillary subtype, which was relevant to survival advantage (Figure 4F).

### Individual Modification Patterns of DNA Methylation

By now, the experimental results have confirmed that DNA methylation is irreplaceable in the formation of distinct TME landscapes. However, investigations above were not helpful to predict the DNA methylation status of an individual sample as they were conducted on a population. Since tumors are heterogeneous and complex, we built the DMRscore model to qualify the DNA methylation status based on the expression of DMRGs.

In this section, we attempted to assess whether DMRscore is effective in predicting clinical outcomes. Patients were classified into groups of high and low DMRscores according to the best cutoff value. The correlation results between DMRscores and clinical outcomes showed that patients in the low DMRscore group exhibited a remarkable clinical advantage, while those in the other group demonstrated less satisfactory clinical outcomes (TCGA-BLCA cohort) (Figure 4E, *p* = 7.158e-05). The Figure 4H shown that patients with high DMRscore were enriched in APC_co_inhibition, T_cell_coinhibition, which revealed that this subgroup presented immunosuppression. Subsequently, we examined whether the DMRscore can serve as an independent index to evaluate the clinical outcomes of bladder cancer. Multivariate Cox regression analysis was used to take the independent indices, including age and DMRscore, into the calculation, and the results confirmed DMRscore as an independent and robust prognostic index (HR = 1.05; Supplementary Figure S4A). Variations of individual patients are displayed by the Sankey diagram (Figure 4F).

**FIGURE5:**
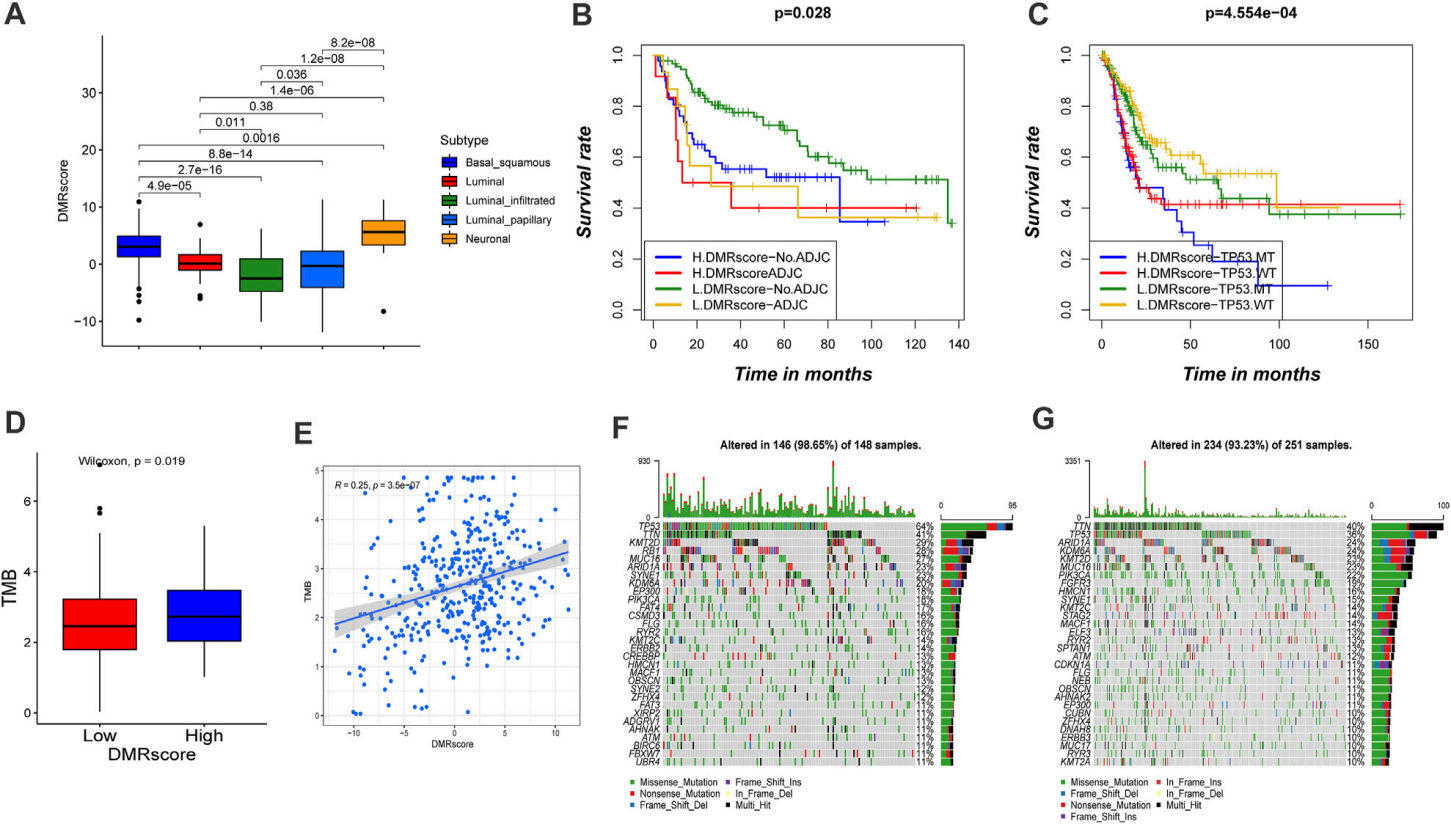
Characteristics of DMRscore in TCGA molecular subtypes and tumor mutation burden. **(A)** Differences in DMRscore among diverse bladder cancer molecular subtypes. Basal squamous subtype, blue; Luminal subtype, red; luminal infiltrated subtype, green; luminal papillary subtype, sapphire; and Neuronal subtype, yellow. **(B)** Kaplan-Meier curve showed the clinical prognosis of patients with combination of DMRscore and adjuvant chemotherapy stratification. H, high. L, low. ADJC, adjuvant chemotherapy (*p* = 0.028). **(C)** Kaplan-Meier curve showed the clinical prognosis of patients with combination of DMRscore and TP53 stratification. H, high. L, low. MT, mutation type; WT, wild type (*p* < 0.001). **(D)** Difference in tumor mutation burden between high DMRscore and low DMRscore (*p* = 0.019). **(E)** Correlation between DMRscore and tumor mutation burden. (R = 0.25, *p* < 0.001) **(F,G)** The landscape of tumor somatic mutation in TCGA-BLCA established by high **(F)** and low DMRscore **(G)**. Each column represented individual patients. The upper barplot displayed tumor mutation burden.

To reassure the predictive effects of DMRscore, we examined its relationship with the identified clusters by Kruskal-Wallis tests. The test results suggested that DMRscore could be used to predict DNA methylation clusters. Specifically, both DMRcluster B and Gene cluster B showed the highest median DMRscore (Figures 4B,C). In addition, patients suffering from neuronal bladder cancer also exhibited the highest median DMRscore among five molecular subtypes (Figure 5A). In a word, DMRscore has been proved as an effective index to assess the DNA methylation status of individual samples and predict clinical outcomes. In order to develop the accuracy of predictive performance, the prognostic nomogram included a DMRscore, and other clinical variables was constructed to evaluate the 1-, 3-, and 5-year overall survival probabilities (Supplementary Figure S6).

**FIGURE 6:**
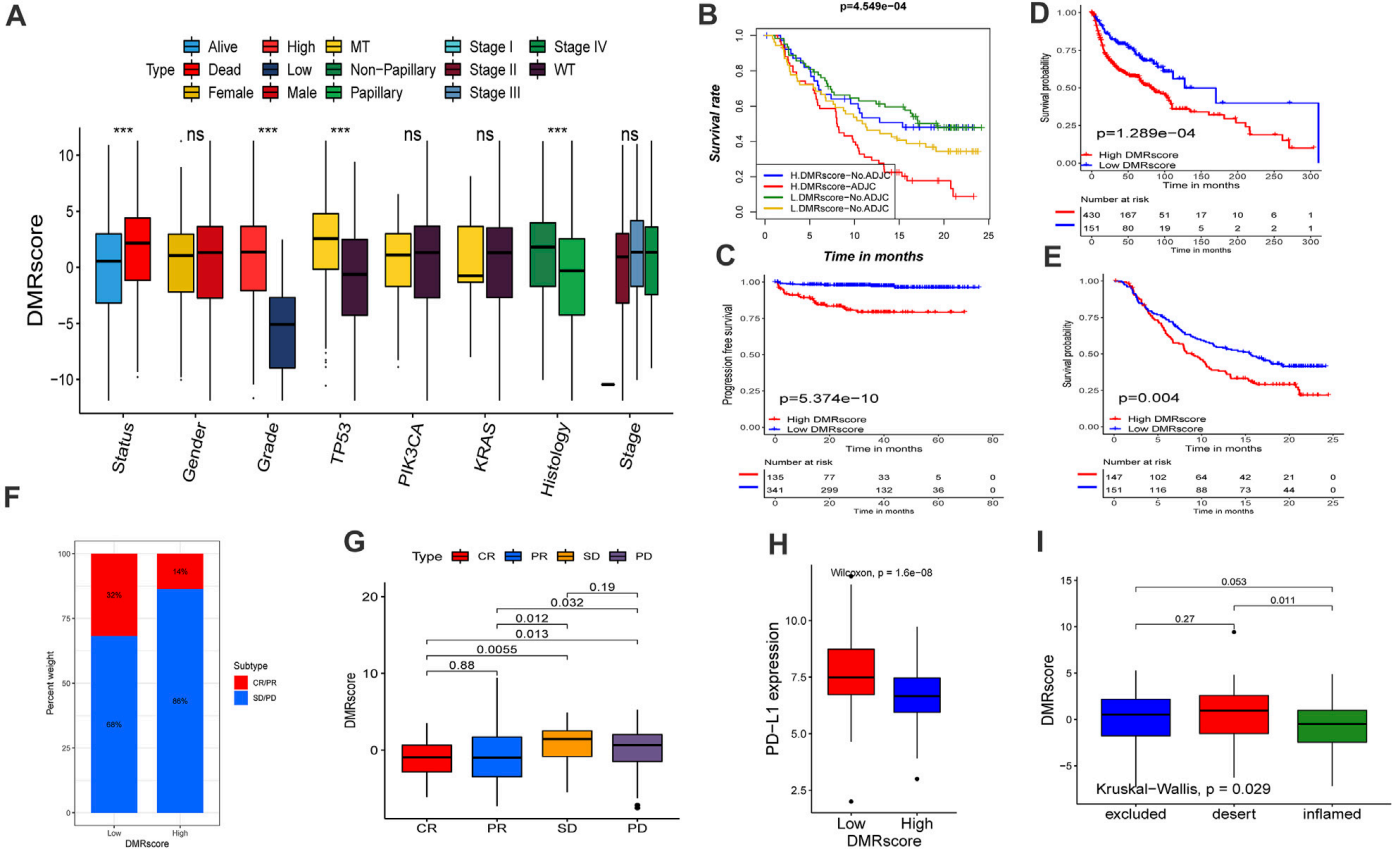
Role of DNA methylation modification in clinical prediction. **(A)** Differences in DMRscore among different clinical status. The median value: black lines in boxes, the outliers: black dots out boxes. MT, mutation type; WT, wild type. **(B)** Kaplan-Meier curve showed the clinical prognosis of patients with combination of DMRscore and NEO stratification. H, high. L, low. NEO, Newantigen burden (*p* < 0.001). **(C)** Survival analyses for high (135 samples) and low (341 samples) DMRscore subgroups in the E-MTAB-4321 cohort using Kaplan-Meier curves (*p* < 0.001). **(D)** Survival analyses for high (430 samples) and low (151 samples) DMRscore subgroups in the GEO-metacohort cohort using Kaplan-Meier curves (*p* < 0.001). **(E)** Survival analyses for high (147 samples) and low (151 samples) DMRscore subgroups in the anti-PD-L1 cohort (IMvigor210 cohort) using Kaplan-Meier curves (*p* = 0.004). **(F,G)** The proportion of patients with response to PD-L1 blockade immunotherapy in low or high DMRscore subgroups. SD, stable disease; PD, progressive disease; CR, complete response; PR, partial response. Responser/Nonresponer: 32%/68% in the low m6Ascore groups and 14%/86% in the high m6Ascore groups. H Differences in PD-L1 expression between low and high m6Ascore groups (*p* < 0.0001). L Differences in DMRscore among distinct tumor immune phenotypes in IMvigor210 cohort. (*p* = 0.029).

Particularly, the capability of DMRscore to assess the efficacy of adjuvant chemotherapy (ADJC) in bladder cancer patients was evaluated. DMRscore prediction results were not disturbed by ADJC: whether receiving ADJC or not, the low-DMRscore group always presented significant survival advantages. However, DMRscore cannot be utilized to judge whether ADJC can be applied on a bladder cancer patient, and patients with low DMRscores had shorter survival after ADJC. (Figure 5B). In addition, patients with high grade, TP53MT, and non-papillary subtypes of the cancer showed significantly higher DMRscores, with a poorer survival prognosis (Figure 6A). This also validated in E-MTAB-4321 cohort (Supplementary Figure S4C). Furthermore, the capability of DMRscore to assess the efficacy of TP53 mutation in bladder cancer patients was examined as well. We found that the L. DMRscore-TP53. WT group exhibited a remarkably advantageous survival, while the H. DMRscore-TP53. MT group demonstrated the worst clinical outcome (Figure 5C). K-M survival analysis and multivariate Cox regression analysis for the E-MTAB-4321 cohort also verified that DMRscore can serve as an independent prognostic index in bladder cancer (Figure 6C; Supplementary Figure S4B).

### The Role of DNA Methylation Mode in anti-PD-1/PD-L1 Immunotherapy

Studies have verified that patients’ response to immunotherapy is related to the TMB frequency, and higher TMB statuses lead to a persistent response to anti-PD-1/PD-L1 immunotherapy. We investigated the somatic mutation frequencies between high- and low-DMRscore groups in the TCGA-BLCA cohort. However, TMB quantification analysis verified that DMRscore is significantly and positively correlated to TMB (Figures 5D,E). Besides, the waterfall diagram showed that the high-DMRscore group was more susceptible to somatic mutations than the other group, with somatic mutation frequencies of 146/148 (98.65%) and 234/251 (93.23%), respectively (Figures 5F,G). Thus, our experimental results indicated that patients with a high DMRscore exhibit good response to anti-PD-1/PD-L1 immunotherapy, which is contradictory to previous findings. Consequently, we speculated that TMB frequency cannot be utilized to predict the effect of immunotherapy in this model.

In order to further examine the prediction performance of the DMRscore model, we applied the established DMRscore signature to other independent bladder cancer cohorts. Almost all cohorts presented survival differences as revealed by the DMRscore model except for two GEO datasets with few samples (Supplementary Figures S5A–E). The prediction performance of the DMRscore model for tumor stages was assessed by the receiver operating characteristic (ROC) curves, and the area under the curve (AUC) was 0.699 and 0.721at 3 and 5 years, respectively (Supplementary Figures S5F,G). These data indicated that the DMRscore signature could serve as a new biomarker to predict clinical outcomes.

Immune checkpoint blockade therapy has undoubtedly produced significant therapeutic benefits for many cancer patients. Based on the collected immunotherapy cohorts, we explored whether DMRscore can serve as a signature to predict patients ' response to immunotherapy. Remarkable survival advantages are seen in patients with low DMRscores in the GEO-metacohort and anti-PD-L1 cohorts (IMvigor210, advantaged urothelial cancer) (Figures 6D,E). In addition, these patients also exhibited much better clinical outcomes when receiving anti-PD-L1 immune checkpoint blockade therapy (Figure 6F), and they were characterized by a significantly higher expression of PD-L1, a potential clinical response to immunotherapy (Figure 6H). Besides, we evaluated the tumor neoantigen burden in bladder cancer patients, and those with high neoantigen burden and low DMRscore signatures presented a significant survival advantage. (Figure 6B). Judging from Figure 6G, the DMRscore signature was a robust and potential biomarker to estimate patient response and clinical outcomes in immunotherapy. The immunophenotypes of metastatic urothelial cancer have been distinguished in the IMvigor210 cohort, so we studied the differences of DMRscore among them (Figure 6I). Most patients with low DMRscores exhibited the inflamed immunophenotype, to which individualized immunotherapy is crucial in treatment. In a word, DNA methylation modes are significantly related to tumor immunophenotypes and patients’ clinical responses to immunotherapy.

## Discussion

DNA methylation is closely related to tumorigenesis and tumor progression. The extent of DNA methylation varies among cancer types and different stages of cancer progression. For example, the progression of prostate cancer has been related to DNA hypomethylation (Fraser et al., 2017; Wu et al., 2020), while bladder cancer pathology was characterized by global DNA hypermethylation (Osei-Amponsa et al., 2020). Thus, this observation revealed that DNA methylation may occur in a cancer-specific manner and alter the tumor microenvironment. Liu P et al. demonstrated that DNMT1 regulated the tumor growth in bladder cancer via modulating the status of DNA methylation in the promoter of PTEN (Liu et al., 2020). Zhu Y et al. demonstrated that MBD2 was a protective signature against bladder carcinoma according to the RNA data from the peripheral blood lymphocytes of 98 bladder cancer patients and 135 frequency-matched control patients (Zhu et al., 2004). Ying L et al. confirmed that epigenetic repression of RGS2 by UHRF1 contributes to bladder cancer progression (Ying et al., 2015). However, most researches only focused on the effect of a single DNA methylation regulator on the alteration of TME and tumor progression. As a result, the landscape of immune cell infiltration characteristics, which is mediated by the synergistic effect of multiple DNA methylation regulators, remained less understood. By clarifying the roles of diverse DNA methylation modes in immune cell infiltration, our knowledge about TME and anti-tumor response could advance, and foundations of more efficient immunotherapy strategies could be established.

In this study, we identified three DNA methylation modes based on expression level 15 DNA methylation regulators, and each DMRcluster was found to correlate with significantly different TMEs. Specifically, DMRclusters A, B, and C are characterized by immune-inflamed, immune-desert, and immune-excluded phenotypes, respectively. The immune-inflamed phenotype, or “hot tumor,” is characterized by the existence of a large number of immune cells in the TME (Zhang et al., 2020b; Gruber et al., 2020; Yu et al., 2021). The other two phenotypes, or “cold tumor,” show non-inflammatory infiltration. Despite the immune-excluded phenotype exhibits considerable immune cell infiltration, the immune cells are constrained by the stromal component that can be present either in the tumor capsule or throughout the whole tumor tissue to prevent the immune cells from exerting anti-tumor effects (Lambrechts et al., 2018; Kaymak et al., 2021). Such an idea is verified by the strong stromal activation in DMRcluster C, where the EMT pathway inhibited the activity of immune cells. Thus, our classifications of different DNA methylation modes were confirmed feasible and effective.

In addition, we confirmed that the transcriptomes in distinct DNA methylation modes are different, and obtained differentially expressed genes among thses DNA methylation patterns. Their actual compositions are related to DNA methylation and immune-related biological pathways. Therefore, we termed these differentially expressed genes as DMRGs. Three genomic subtypes were divided from the samples based on the expression of DMRGs, and these subtypes were also significantly related to distinct immunophenotypes. Therefore, DNA methylation is indeed irreplaceable in shaping the TME, and a systematic assessment of DNA methylation modes will contribute to understanding the mechanisms of tumorigenesis and to the advancements of personal medicine.

Since tumors are heterogeneous, we built a DMRscore model to evaluate DNA methylation features in individual tumors. The patients with high DMRscores were characterized as the immune-desert phenotype, while the patients with low DMRscores were characterized as the immune-inflamed phenotype. These results were further verified in the IMvigor210 cohort whose immunophenotypes have been identified (Necchi et al., 2017). Comprehensive analyses suggest that DMRscore signature is a robust and potential biomarker to assess patients’ response to immunotherapy, Patients with low DMRscore displayed higher expression of PD-L1 compared to patients with higher DMRscore, and had a better response to Atezolizumab. In addition, patients with low DMRscores exhibited less TP53 wild mutation, lower cancer grade, low tumor mutation burden, and molecular subtypes were mainly papillary subtypes.

In summary, DMRscore can systematically assess the DNA methylation landscape and detect the TME characteristics, thereby identifying the immunophenotypes of individual patients for more efficient immunotherapeutic strategies. Besides, DMRscore can be used to evaluate other features of bladder cancer patients, including molecular subtypes, genetic mutation, tumor stage, and clinical histology. Moreover, DMRscore could serve as an independent prognostic indicator for effective prediction of clinical outcomes, as well as a factor that reflects the efficacy of and clinical responses to immunotherapy. Our research uncovers that DNA methylation can alter the immune microenvironment, resulting in the emergence of a “cold tumor.” Herein, we propose a new hypothesis: targeting DNA methylation regulators or DMR-related biological pathways could be effective to alter the DNA methylation status so that the unfavorable factors could be removed, and “cold tumors” could transform into “hot” ones. If proved correct, this hypothesis may promote the development of immunotherapeutic agents and drug combinations. Our study provided a new perspective to reveal the global DNA methylation status in bladder cancer patients, to predict the immunophenotype of individual tumors, and to promote individualized medicine.

Compared with existing investigations on prognostic signatures of bladder cancer, this study has some noteworthy advantages and shortcomings. Firstly, our investigation contributed to demonstrate the effect of DNA methylation modification in shaping of tumor microenvironment complexity and diversity, and explored the potential role of DNA methylation status to predict the clinical response to Atezolizumab therapy in urothelial carcinoma. The global DNA methylation landscape was constructed as the observation object to systematically investigate the effect of DNA methylation modification on tumor microenvironment, which has not been clarified before this study. Our study is mainly based on bioinformatics analysis and requires further clinical verification. Basic experiments are needed to verify the relationship between prognostic characteristics and immune infiltration; In the future we will conduct multicenter, large sample size studies to prospectively validate the model in order to further test the predictive potential and clinical ability of our model.

## Methods

### Data Acquisition and Processing

The workflow in our study is displayed in Supplementary Figure S1A. 7 sets of transcriptome data and their corresponding clinical annotations were obtained from The Cancer Genome Alta (TCGA) and Gene-expression omnibus (GEO) databases, in which patients without complete clinical annotation were excluded. The “ComBat” algorithm in the R package “sva” was used to correct the batch effect of non-biological technical deviations. The comprehensive information of all alternative bladder cancer datasets is summarized in Table 1. The transcriptome data were downloaded from UCSC Xena database, and the somatic mutation information was obtained from TCGA database. We investigated numerous DNA methylation regulators, including the DNA methyltransferase family (DNMT1, DNMT3A, DNMT3B), the methyl-CpG-binding domain proteins (MeCP2, MBD1, MBD2, MBD3, MBD4), the ubiquitin-like proteins containing PHD and RING finger domains (UHRF1, UHRF2), zinc-finger domain proteins (ZBTB33, ZBTB4), NTHL1, SMUG1, and UNG (Jones, 2012; Moore et al., 2013; Koch et al., 2018). All the data were processed with the R package “Bioconductor” in R software (version 4.0.3).

**TABLE 1:** The gene expression profiles of bladder cancer included in this study.

Accession number	Source	Number of patients	Survival
TCGA: BLCA	Illumina RNAseq	432	OS
GEO: GSE13507	Illumina human-6 v2.0 expression beadchip	256	OS
GEO: GSE31684	Affymetrix Human Genome U133 Plus 2.0 Array	93	OS
GEO: GSE32548	Illumina HumanHT-12 V3.0 expression beadchip	131	OS
GEO: GSE48075	Illumina HumanHT-12 V3.0 expression beadchip	142	OS
GEO: GSE48276	Illumina HumanHT-12 WG-DASL V4.0 R2 expression beadchip	116	OS
GEO: GSE70691	Illumina HumanHT-12 WG-DASL V4.0 R2 expression beadchip	49	OS
ArrayExpress: E-MTAB-4321	Illumina HiSeq 2000	476	PFS
IMvigor210	Illumina RNAseq	348	OS

### Unsupervised Clustering of the Fifteen DNA Methylation Regulators

The unsupervised clustering algorithm was utilized to find out the distinct DNA methylation patterns based on the expression of the fifteen alternative DNA methylation regulators. The R package “ConsensuClsterPlus” was run 1,000 repetitive times to ensure the stability of classification, and the clustering number was assigned according to the K value. Subsequently, to verify the differences in biological functions among the three DNA methylation patterns, we ran the R package “GSVA.” GSVA (gene set variation analysis) is an unsupervised method to evaluate variations of biological pathways in a sample population (Hänzelmann et al., 2013). The gene sets identified from GSVA were named “c2.cp.kegg.v6.2.-symbols.”

### Estimation of Tumor Microenvironment Cell Infiltration

The ssGSEA (single-sample gene set enrichment analysis) was utilized to calculate the relative amounts of gene components in each TME cell infiltration. A gene set that labels each immune cell type was adopted from the published studies (Charoentong et al., 2017). We investigated several immune cell types, including activated B cells, activated CD4 T cells, macrophages, eosinophils, CD56dim natural killer cells, and neutrophils. Each type of immune cell was counted for its enrichment score, and a box diagram was used to compare the scores in different DNA methylation patterns.

### Identification of DNA Methylation Related Genes Among Distinct DNA Methylation Modes

To reveal which genes are DMRGs, we classified the samples into three DNA methylation clusters based on the expression levels of fifteen DNA methylation regulators. The differentially expressed genes (DEGs) among the clusters were picked out by an R package named “limma” with the adjusted *p*-value <0.001.

### Construction of DNA Methylation Regulator Score Groups

To qualify the DNA methylation status of individual samples from bladder cancer patients, we designed a DNA methylation signature termed DMRscore for assessments. The DNA methylation score groups were designed as follows: After identifying the DMRGs, we extracted the overlapping genes in them. Patients were divided into groups with distinct immune subtypes for further analysis, which was performed by an unsupervised clustering approach on the overlapping DMRGs. The number of gene clusters and model stability were validated by the consensus clustering algorithm. Furthermore, the univariate Cox regression analysis was executed for each overlapping DMRG to find out the ones related to prognosis. Subsequently, the established DMRscore model was subject to the principal component analysis (PCA) that combines the linear high-dimension indicators into their linear independent low-dimension counterparts. Moreover, in PCA, both types of indicators retain their original information, and the speed of data processing is accelerated. Both principal components (i.e., the two types of indicators) were extracted to calculate the DMRscore as DMRscore = ∑(PC1i + PC2i), where i is the expression level of the prognostic-related DMRGs.

To comprehensively evaluate the clinical outcome of each patient, a prognostic nomogram that contained the T stage, M stage, N stage, Gender, Age, clinical Stage and DMRscore was constructed. Subsequently, the 1-, 3-, 5- year overall survival probabilities were assessed by the calibration curve. A calibration curve close to 45° indicated the prominent prediction ability of the constructed model.

### The Relationship Between DNA Methylation Features and Other Relevant Biological Functions

We obtained several gene sets that are involved in certain biological processes, e.g., DNA damage repair, homologous recombination, cell cycle, mismatch repair, DNA replication, nucleotide excision, carcinogenesis, Pan-F-TBRS, EMT, angiogenesis, immune checkpoint, actions of CD8 T effector cells, and antigen processing (Rosenberg et al., 2016; Şenbabaoğlu et al., 2016; Mariathasan et al., 2018). The correlations between DNA methylation features and these processes were further identified via correlation analysis.

### The Genomic Profiles of Immune Checkpoint Blockage Effects and Corresponding Clinical Information

In order to explore the predictive effect of DNA methylation statuses in immunotherapy, we included an immunotherapeutic cohort in this study, advanced urothelial cancer treated with atezolizumab (IMvigor210 cohort) (Rosenberg et al., 2016). Atezolizumab is anti-PD-1 monoclonal antibody. The transcriptome profiles of immune checkpoint blockage effects and their corresponding clinical information were obtained from the public dataset.

### Statistical Analysis

Data processing was conducted solely on R software (version 4.0.3). The R package named “limma” was run to analyze differential gene expressions among distinct subtypes. The Spearman analysis and distance correlation analysis were performed to calculate correlation coefficients between the DNA methylation regulators and the infiltration of immune cells. The survival curves of bladder cancer patients were plotted via the Kaplan-Meier method, and the curves’ area under the curve (AUC) was calculated to evaluate the specificity and sensitivity of DMRscores obtained by the R package “pROC”. The location and circle sequence of the DNA methylation regulators along the chromosomes were depicted by the R package “RCircos”. Moreover, the R package “DEseq2” was run to normalize the raw data and convert the normalized cell count to TPM in the “IMvigor 210” cohort. Finally, the mutation landscape was drawn and presented via the R package “maftools.” All statistic p numbers were bilateral, and *p* < 0.05 was considered statistically significant.

## Data Availability

All datasets generated for this study are included in the article material, including the TCGA. database (https://portal.gdc.cancer.gov/), and GEO dataset (https://www.ncbi.nlm.nih.gov/gds/).
