# Supplementary material for: DNA Methylation Modification Map to Predict Tumor Molecular Subtypes and Efficacy of Immunotherapy in Bladder Cancer
Source: Front Cell Dev Biol. 2021 Dec 3;9:760369. doi: 10.3389/fcell.2021.760369 (PMC8678484; doi:10.3389/fcell.2021.760369)
Supplement: Supplementary file 1 [file DataSheet1.docx]

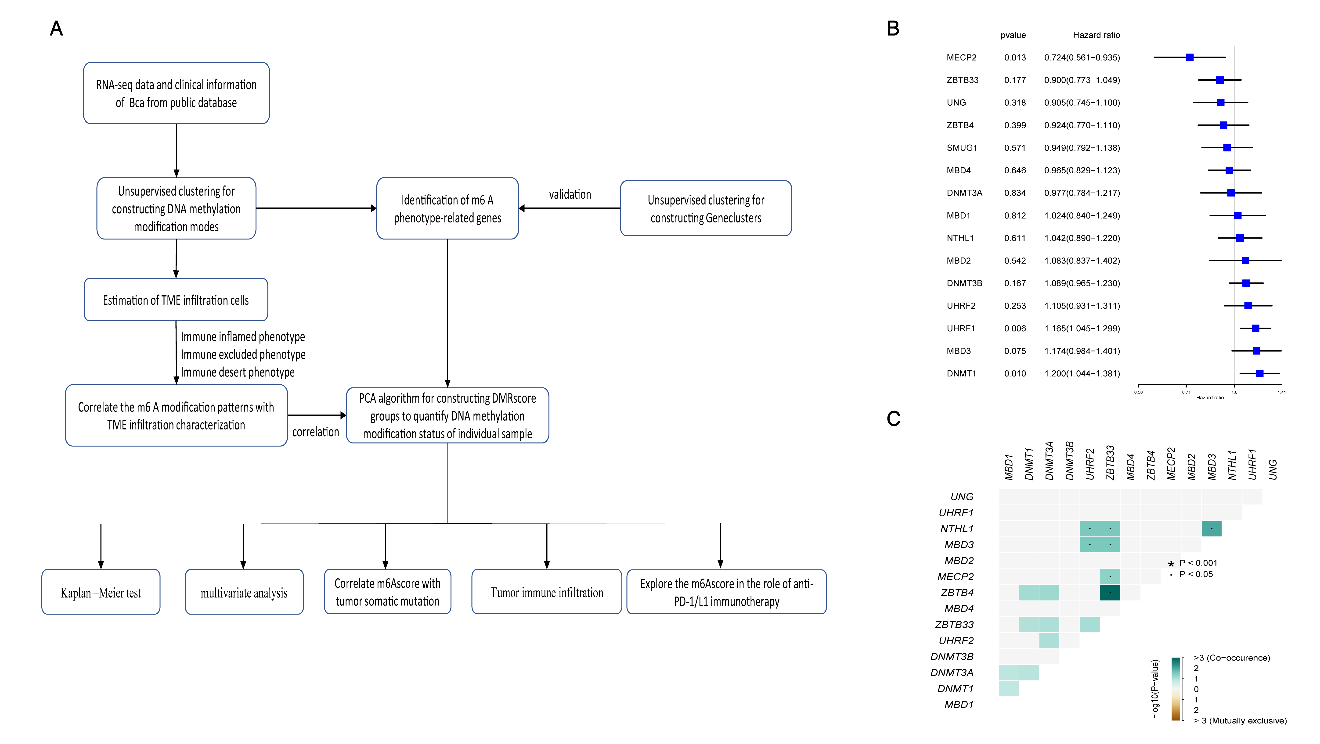


Figure S1| The research design and prognostic analysis of 15 DNA methylation regulators. A The flow chart of this article. B The prognostic analyses for 15 DNA methylation regulators via using the univariate Cox regression model. C The mutation exclusion and co-occurrence analyses for 15 DNA methylation regulators. Co-occurrence, green; Exclusion, yellow.


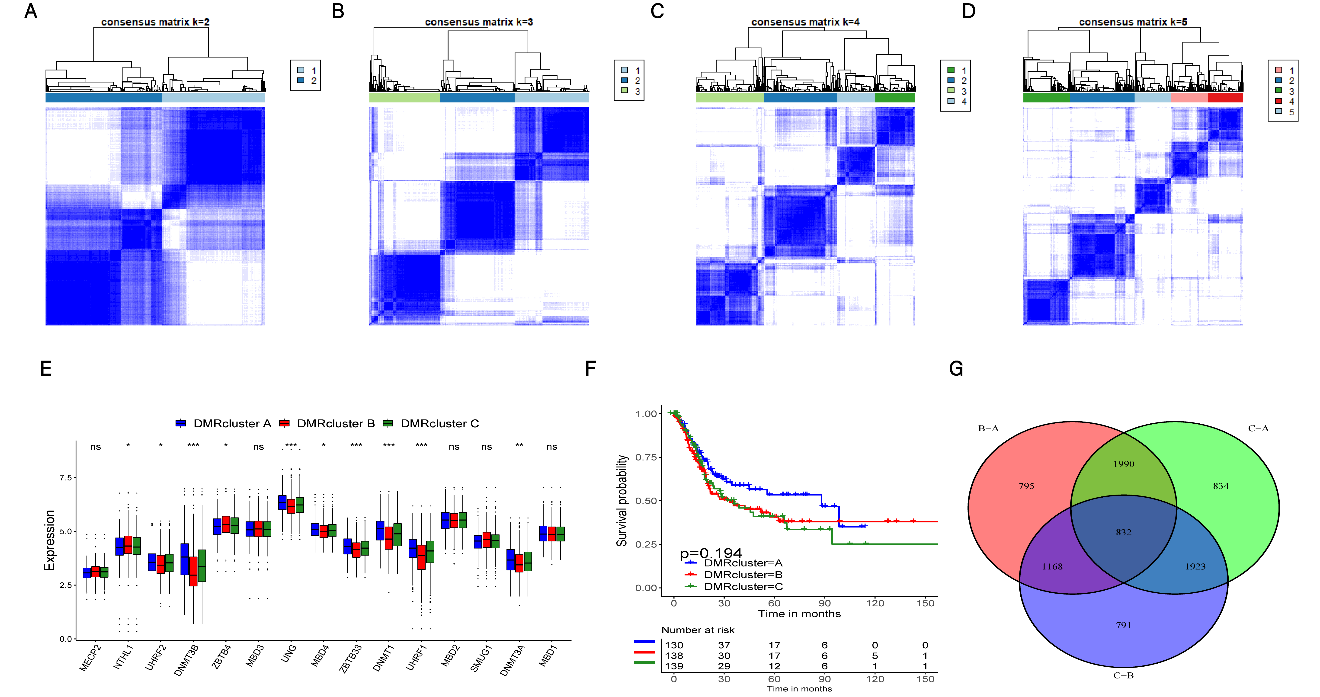


Figure S2|(A-D) Unsupervised clustering of DNA methylation regulators in TCGA cohort and consensus matrices for k = 2 - 5. (E) Difference in the expression of known signatureConsensus matrix of the TCGA cohort. k = 2 - 5. (E) The expression of 21 m6 A regulators in the three m6Aclusters. The median value: black lines in boxes, the outliers : black dots out boxes. (F)  Kaplan-Meier curve with *p* value 0.194 displayed a difference among three DNA methylation modes in TCGA-BLCA cohort. DMRcluster A: 130 samples, DMRcluster B: 138 samples and DMRcluster C: 139 samples. (G) 832 DNA methylation related genes shown in venn diagram.


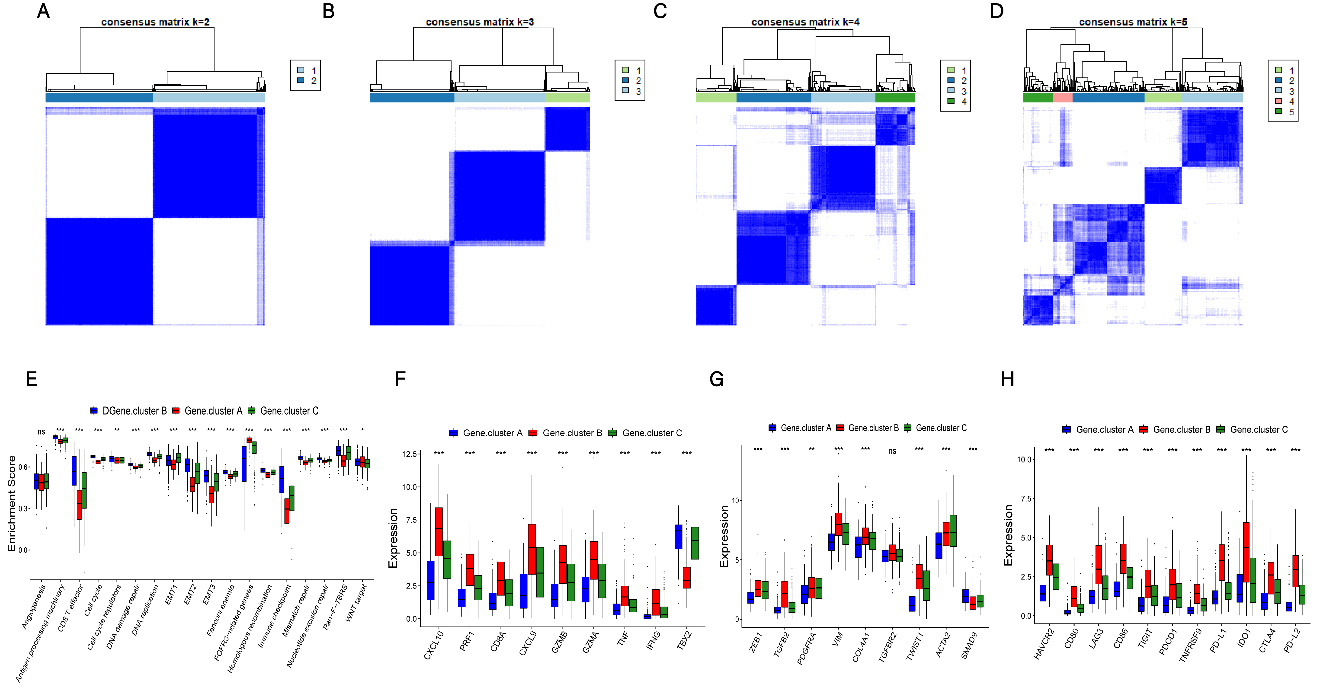


Figure S3|(A-D) Consensus matrix of the TCGA cohort (Geneclusters). k = 2 - 5. (E-H) The Difference of known signals among three Gene.clusters, including stromal/immune activation signals, tumor progression signals and immune checkpoint related signals. The median value: black lines in boxes, the outliers : black dots out boxes. The asterisks represented the statistical p value.(*P < 0.05; **P < 0.01; ***P < 0.001)


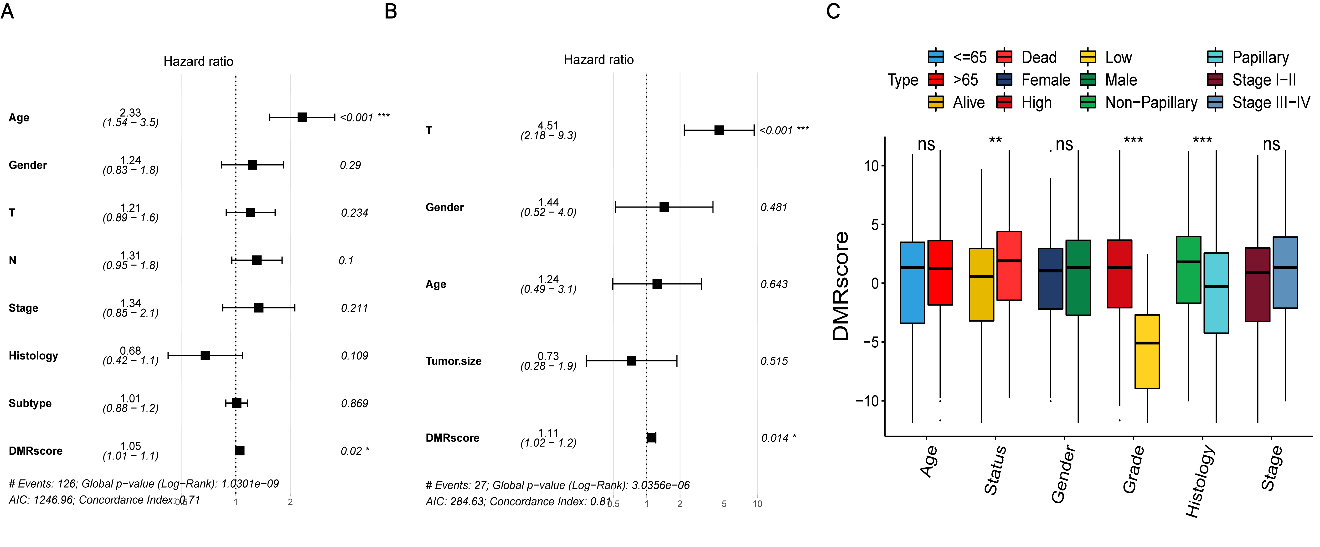


Figure S4| The prognostic assessment of DMRscore. (A-B). Multivariate Cox regression analysis for DMRscore in TCGA-BLCA cohort(A) and E-MTAB-4321 cohort (B) were displayed by the forest diagrams. C.  Differences in DMRscore among different clinical status. The median value: black lines in boxes, the outliers : black dots out boxes.


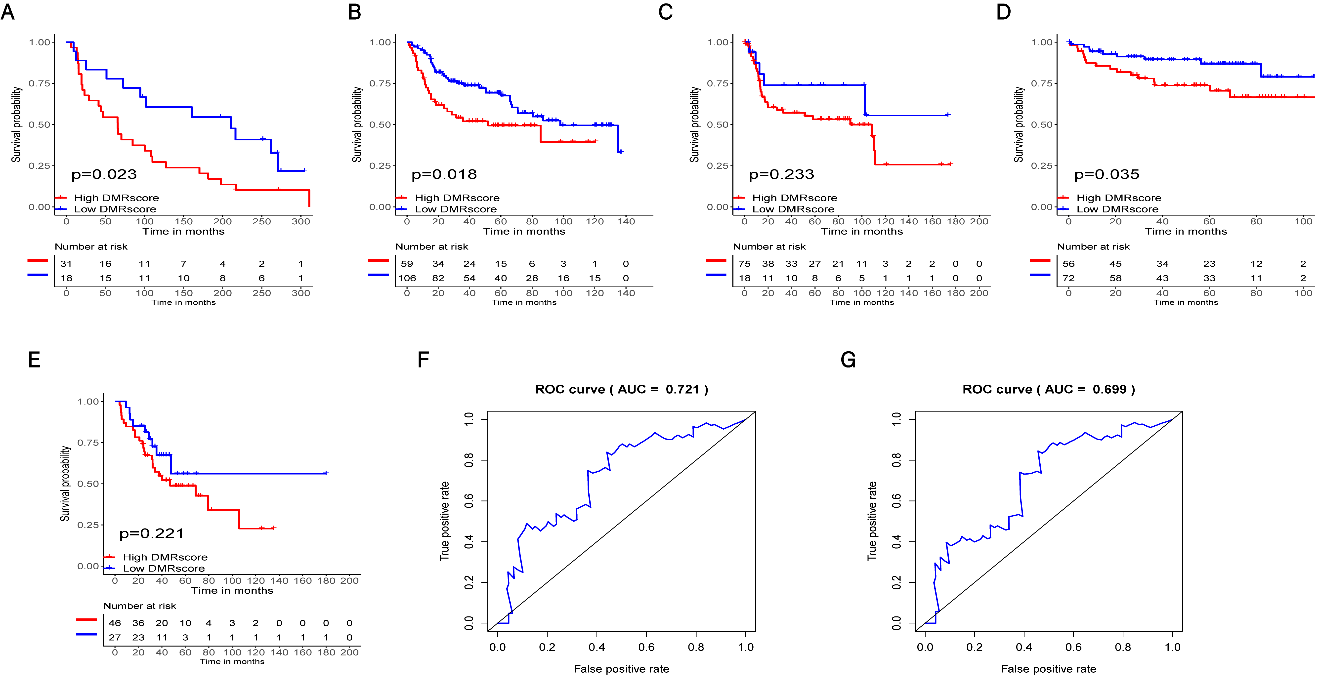


Figure S5| (A-F) Kaplan-Meier curve showed the clinical prognosis of patients with high- and low-DMRscore in different cohorts(GSE70791, GSE13507, GSE31684, GES32548, GES48276 respectively) . (F-G) Predictive value of DNA methylation quantification in stage 12 patients for 3 and 5 years (TCGA-BLCA cohort).


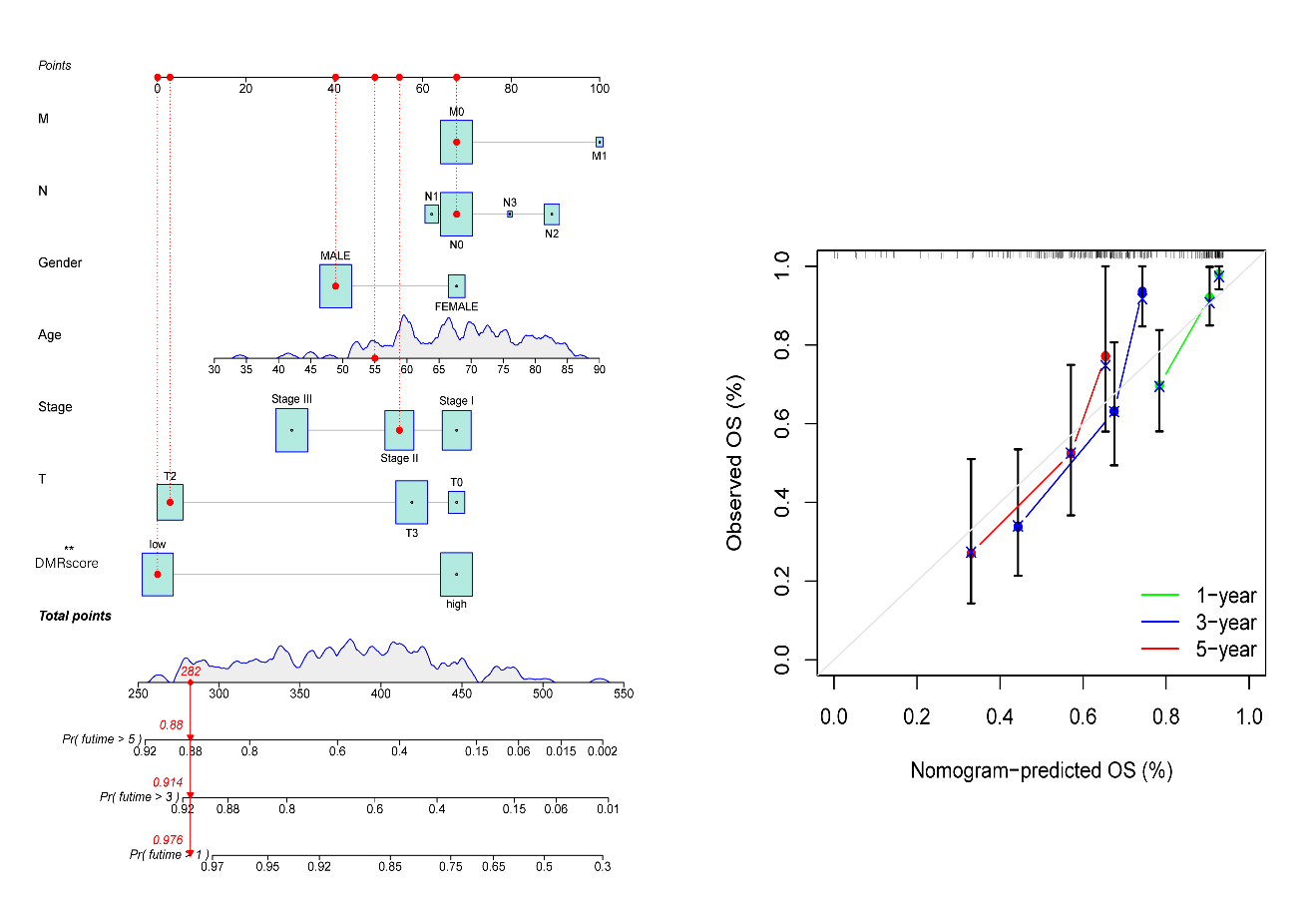


Figure S6|(A)Nomogram established by T stage, M stage, N stage, Gender, Age, clinical Stage and DMRscore for predicting overall survival probability of bladder cancer patients. (B) Calibration curve of 1‐, 2-, 3-year nomogram, the predicted performances of the model are represented by the 45º gray lines. The green/blue/red line represents 1/3/5 years prediction ability.
